# Diabetes and atrial fibrillation: stratification and prevention of stroke risks

**DOI:** 10.1186/1878-5085-5-17

**Published:** 2014-10-03

**Authors:** Stephan v Bandemer, Sebastian Merkel, Anna Nimako-Doffour, Mattias M Weber

**Affiliations:** 1 Institute of Work and Technology, Munscheidstr 14, 45886 Gelsenkirchen, Germany; 2 Universitätsmedizin Mainz der Johannes Gutenberg-Universität, Langenbeckstraße 1, 55131 Mainz, Germany

**Keywords:** Diabetes, Atrial fibrillation, Stroke risk, Personalized prevention strategies

## Abstract

**Background:**

Although evidence is not very clear, diabetes is assumed to be an independent risk factor for atrial fibrillation (AF). One reason for the lack of evidence could be that AF often is not detected due to its paroxysmal or asymptomatic character. A better understanding of the relationship between both diseases and improved detection of AF is necessary since the combination of both diseases dramatically increase the risk of strokes if not treated properly.

**Methods:**

Available literature about diabetes as an independent risk factor for AF has been evaluated, and limitations of studies are discussed.

**Results:**

Results from different trials and registers are contradictory concerning diabetes as an independent risk factor for AF. Reasons for these differences can be found in different study designs and neglecting patients with unknown AF.

**Conclusions:**

Due to the increasing burden of disease of diabetes and AF as common risk factors for stroke, a systematic screening for AF in diabetes patients could provide a better understanding of their correlation and personalized prevention strategies.

## Overview

### Predictive, preventive and personalized medicine in the case of diabetes and AF

Diabetes and *predictive, preventive and personalized medicine* (PPPM) have been the focus of EPMA since its inception [1]. Especially, biomarkers for the prediction of diabetes complications [2-4], specific complications like diabetic retinopathy [5] or cancer [6] as well as questions of drug delivery [7,8] have been addressed by the research. The first EPMA World Congress in 2011 addressed public health issues, diabetes education and lifestyle changes and dealt with genomics, proteomics and metabolomics identifying individuals at risk of developing the disease [9].

At the second EPMA World Congress in 2013, among other topics, problems of multimorbidity of diabetic patients were further stressed [10]. This raises the question whether diabetes has to be considered as an independent risk factor for other diseases (e.g. atrial fibrillation (AF)) and how the combination of diseases with different etiologies increase the risk of severe complications like heart diseases and stroke. This paper addresses the question of the interdependency between diabetes and AF as a major stroke risk and discusses research requirements in order to advance the understanding of multimorbidity in diabetes patients as a prerequisite of appropriate prevention strategies.

### Epidemiology of diabetes and atrial fibrillation

Diabetes is one of the biggest epidemiological challenges worldwide. According to the International Diabetes Federation, 366 million people suffered from diabetes type 1 and type 2 in 2011. By 2030, this number will increase to 552 million people. Diabetes caused 4.6 million deaths in 2011 and at least 465 billion dollars of health care expenditures, being responsible for 11% of total health care expenditures [11].

Diabetes-associated AF is a chronic disease with high prevalence and a progressive increase in its severity which is associated with a high risk of stroke, heart failure and death [12,13]. Based on the analysis of the Rotterdam cohort study [14], the total prevalence of AF is 8.6 in men and 7.1 in women. The prevalence increases with age from 1.3/1.7 in the population between 55 and 59 years to 24.2/16.1 in the population above 85 years. Projected age- and sex-adjusted prevalence for the European Union will more than double from 8.8 Mio. in 2010 to 17.9 Mio. in 2060 [15].

The need for better prevention and treatment of diabetes and AF is complicated by a lack of knowledge about diabetes as an independent risk factor for AF and problems in detecting AF as a risk factor for stroke especially in combination with diabetes. Therefore, the analysis of the multimorbidity of patients is an important task in order to stratify risks and risk prevention strategies for individual patients.

## Methods

An evaluation of available literature, trials and registers about diabetes as an independent risk factor for AF shows contradictory results. Possible reasons for these differences are discussed, and options for overcoming limitations of existing studies are debated. Limitations of the available literature are that there are no studies available which are based on AF screening in patients with diabetes.

## Results

### Diabetes as an independent risk factor for AF and associated stroke risk

The evaluation of literature about the relation of diabetes as an independent risk factor for AF can be based on different types of studies. Some registers and epidemiological studies provide quantitative data about the relation of diabetes and AF [15-19]. This literature has its limitations especially in a lack of adjustments of common risks of diabetes and AF and does not provide details about the diseases. Other studies additionally take common risk factors of diabetes and AF as well as diabetes duration and glucose control into account [20,21]. These studies however do not address detection problems of AF and seem to underestimate prevalence. Some studies are available about the detection of unknown AF in the case of (cryptogenic) strokes, but they do not analyze the relation to diabetes systematically [22-24].

Large registries like RE-LY AF show that the prevalence of diabetes in patients with AF is high (21.8) [15,16]. However, the age-adjusted prevalence does not seem to be significantly higher than that in the overall population [25]. These epidemiological data cannot provide reasonable evidence for diabetes as an independent risk factor for AF. Many studies have analyzed just this relation of AF to diabetes or elevated blood glucose as independent risk factors, but results are contradictory. The Framingham Heart Study mentions diabetes as an independent risk factor for AF [17], but the Framingham risk score for AF only confirms known risk factors for AF such as age, sex, body mass index, blood pressure and cardiovascular disease, not diabetes however (OR 1.10; CI 0.87–1.38) [18]. Other studies see an association between diabetes and AF for women but not for men [19].

One reason for the different results could be that both diseases have similar risk factors like age or obesity. Also, paroxysmal AF might be neglected due to detection problems. A study that adjusted age and BMI risks and included all kinds of AF reflecting especially on the role of diabetes duration and glycemic control found an elevated AF risk with diabetes duration and poor glycemic control [21]. In this study, people with diabetes treatment had an overall 40% increased risk for AF compared to those without diabetes (95% CI 1.15–1.71). The risk for AF increased by 3% for every year of diabetes duration (95% CI 1%–6%) and was higher in patients with poor glycemic control. The NAVIGATOR trial analyzed incident AF in patients with no AF in their medical history. It showed that patients with impaired glucose tolerance but no progression to diabetes had a 33% (CI 1.11–1.59) risk increase for AF per 1 mmol/L fasting plasma glucose. However, this trial could not confirm the progression of diabetes as an independent risk factor for AF (HR 0.98, CI 0.80, 1.20) [21]. Thus, there exists at least some evidence for an elevated risk of AF in patients with glucose intolerance or diabetes, but there also are quite some contradictions between different trials.

A limitation of studies about the relation of diabetes and AF is undiagnosed AF (silent AF) since it often is asymptomatic even in the case of permanent AF. Also, in many cases, AF is paroxysmal and difficult to detect [26]. In 15% to 25% of patients with stroke, AF is first detected at the time of stroke incident [22]. And even at stroke incident, AF is often not detected. About 30% of strokes remain cryptogenic [27]. A recent study using automated continuous ECG monitoring during stroke unit stay detected AF in 13.7% of patients who did not have known AF episodes in their medical history and not at admission ECG. Of these patients, 24.6% also had diabetes [23]. In the Cryptogenic Stroke and Underlying Atrial Fibrillation (CRYSTAL-AF) trial which traced 220 patients with cryptogenic stroke by an implantable loop recorder, 29 cases of AF have been detected. The mean time for detection was 84 days [24]. This indicates that AF and the related stroke risks are easily underestimated which might distort results of trials.

## Expert recommendations

Considering that diabetes as well as AF goes along with an approximately up to fourfold stroke risk each [28,29] and that there is some evidence for an increase of AF in diabetic patients, prevention strategies addressing the multimorbidity of the diseases are of substantial relevance. There are gaps in the knowledge about subgroups of patients according to age, sex, ethnicity and socioeconomic status. A subgroup analysis of patients will be necessary in order to personalize prevention strategies. Research also has to focus on the interrelationship between diabetes and AF and common risk factors such as age and obesity which can predispose to these conditions.

### Primary prevention

Since the risk of stroke increases considerably for diabetic patients even with undetected AF or AF without symptoms [30], it will be of high relevance to screen diabetic patients for all kinds of AF systematically. Some cases may be detected by conventional 12-lead ECG, but this will miss those patients with paroxysmal AF and no episode during ECG. The use of Holter ECG will also miss many patients in the case of paroxysmal AF. The CRYSTAL-AF study therefore suggests the use of loop recorders for such screening even in primary prevention [24]. However, due to high costs and the interventional character, economic reasons as well as patient’s acceptance will limit this approach. A promising alternative would be the automated ECG that has provided high specificity and significance in patients with cryptogenic stroke [23].

In order to stratify individual risks of patients with diabetes without a medical history of AF, a screening for AF would at least be appropriate in the case of one additional risk factor according to the CHADS2 score since these patients will definitely profit from oral anticoagulation (OAC) considerably [31,32]. But even diabetic patients without additional risk factors should be eligible for participating in an AF screening since individual decisions for antiplatelet or OAC will have to be taken. This will also provide the opportunity for a stratification of diabetic patients who will rather benefit from treatment with new oral anticoagulants (NOACs) and those who will rather need treatment with vitamin K antagonists. Many diabetes patients will have contraindication for NOACs due to co-morbidities like impaired kidney function and therefore need treatment with warfarin. Co-morbidities like chronic wounds will also require a therapeutic management that coordinates various risk factors and their treatment.

In addition to the preventive purpose of screening diabetic patients for AF, this will provide an increasing understanding of the interrelationship between diabetes and AF. All studies on diabetes as an independent risk factor for AF have focused on patients with known AF and therefore are systematically underestimating asymptomatic and paroxysmal AF.

### Secondary and tertiary prevention

Although there have been some studies concerning secondary prevention especially concerning cryptogenic stroke [23,24], strategies of detection still require further research. The trials have been performed with small numbers of patients, and the stratification of diagnostic instruments needs further validation. This concerns the combination of automated ECGs and implantable loop recorders. Trials also usually have concentrated on one possible reason for cryptogenic stroke but did not address problems of multimorbidity like diabetes, hypertension and AF as possible combined causes.

In order to provide appropriate secondary prevention strategies, cryptogenic strokes need to be analyzed with respect to multiple causes. Personalized strategies to prevent recurrent strokes will have to address different independent risk factors and their interactions. This requires further research into interdependencies like between diabetes and AF. Secondary and tertiary prevention of all stroke patients require consequent follow-up in order to prevent recurrent stroke. This includes lifestyle changes and consequent control especially of glucose levels, anticoagulation, blood pressure and cholesterol levels [33].

### Research networks under the umbrella of EPMA

**Figure 1:**
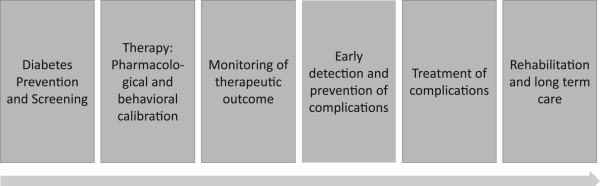
Supply chain of diabetes care.

In order to cope with the increasing multimorbidity of diabetes patients, it will be necessary to organize multidisciplinary teams including different medical specialties as well as industrial partners. Research needs to be organized in a translational way including basic research about the mechanisms of interrelationship between diabetes and AF, screening of AF, prevention of risk factors and the management of complications. EPMA can provide the platform of research for individualized strategies by cooperating with research teams from basic research to the design of health service strategies and patient organization involvement.For this purpose, the cooperation with providers in health clusters will be a promising strategy for the design of health research and treatment strategies. Such clusters can provide research and services along the supply chain (Figure 1) of diabetes care and integrate the different approaches. Utilizing clusters as a basis for international research networks will enable networks for systematically dealing with multimorbidity and translational strategies as a major challenge for PPPM.

### “Horizon 2020” as a powerful instrument to promote the innovation in the field

As it has been reported in its White Paper 2012 as the fundamental strategic document [9], EPMA has strongly contributed to the PPPM-related topics considered within the new European programme “Horizon 2020”. The tasks related to the prediction, prevention and personalized treatments of persons affected by diabetes and its complications and co-morbidities are crucial for the scientific progress in a spectrum of medical fields and health care as the whole. “Horizon 2020” creates a robust platform for the multi- and interdisciplinary professional collaboration as the clue to the dramatic improvements in the pre/diabetes care. Consequently, the professional consolidation in the field should be performed utilizing all the comprehensive instruments systematically provided over the entire duration of the European programme (2014–2020). The complete overview of the strategies and instruments of the “Horizon 2020” is provided by the “Predictive, Preventive and Personalised Medicine as the hardcore of ‘Horizon 2020’: EPMA position paper” [34]. In particular, the below listed calls 2014–2015 might be useful to promote diabetes-related international collaboration, innovative research and advanced health care^a^:

1. Understanding health, ageing and disease

PHC 1—2014: Understanding health, ageing and disease: determinants, risk factors and pathways

PHC 2—2015: Understanding diseases: systems medicine

PHC 3—2015: Understanding common mechanisms of diseases and their relevance in co-morbidities

2. Effective health promotion, disease prevention, preparedness and screening

PHC 4—2015: Health promotion and disease prevention: improved inter-sector cooperation for environment and health-based interventions

PHC 5—2014: Health promotion and disease prevention: translating “omics” into stratified approaches

PHC 6—2014: Evaluating existing screening and prevention programmes

PHC 7—2014: Improving the control of infectious epidemics and foodborne outbreaks through rapid identification of pathogens (see also societal challenge 2)

PHC 8—2014: Vaccine development for poverty-related and neglected infectious diseases: tuberculosis

PHC 9—2015: Vaccine development for poverty-related and neglected infectious diseases: HIV/AIDS

3. Improving diagnosis

PHC 10—2014: Development of new diagnostic tools and technologies: in vitro devices, assays and platforms

PHC 11—2015: Development of new diagnostic tools and technologies: in vivo medical imaging technologies

PHC 12—2014/2015: Clinical research for the validation of biomarkers and/or diagnostic medical devices

4. Innovative treatments and technologies

PHC 13—2014: New therapies for chronic non-communicable diseases

PHC 14—2015: New therapies for rare diseases

PHC 15—2014/2015: Clinical research on regenerative medicine

PHC 16—2015: Tools and technologies for advanced therapies

PHC 17—2014: Comparing the effectiveness of existing health care interventions in the elderly

PHC 18—2015: Establishing effectiveness of health care interventions in the paediatric population

5. Advancing active and healthy ageing

PHC 19—2014: Advancing active and healthy ageing with ICT: service robotics within assisted living environments

PHC 20—2014: Advancing active and healthy ageing with ICT: ICT solutions for independent living with cognitive impairment

PHC 21—2015: Advancing active and healthy ageing with ICT: early risk detection and intervention

6. Integrated, sustainable, citizen-centred care

PHC 23—2014: Developing and comparing new models for safe and efficient, prevention-oriented health and care systems

PHC 24—2015: Piloting personalized medicine in health and care systems

PHC 25—2015: Advanced ICT systems and services for integrated care

PHC 26—2014: Self-management of health and disease: citizen engagement and mHealth

PHC 27—2015: Self-management of health and disease and patient empowerment supported by ICT

PHC 28—2015: Self-management of health and disease and decision support systems based on predictive computer modelling used by the patient him or herself

PHC 29—2015: Public procurement of innovative eHealth services

7. Improving health information, data exploitation and providing an evidence base for health policies and regulation

PHC 30—2015: Digital representation of health data to improve disease diagnosis and treatment

PHC 31—2014: Foresight for health policy development and regulation

PHC 32—2014: Advancing bioinformatics to meet biomedical and clinical needs

PHC 33—2015: New approaches to improve predictive human safety testing

PHC 34—2014: eHealth interoperability

Co-ordination activities:

HCO 1—2014: Support for the European Innovation Partnership on Active and Healthy Ageing

HCO 2—2014: Joint Programming: Coordination Action for the Joint Programming Initiative (JPI) “More Years, Better Lives - the Challenges and Opportunities of Demographic Change”

HCO 3—2015: Support for the European Reference Networks: efficient network modelling and validation

HCO 4—2014: Support for international infectious disease preparedness research

HCO 5—2014: Global Alliance for Chronic Diseases: prevention and treatment of type 2 diabetes

HCO 6—2015: Global Alliance for Chronic Diseases: 2015 priority

HCO 7—2014: ERA-NET: establishing synergies between the Joint Programming on Neurodegenerative Diseases Research and Horizon 2020

HCO 8—2014: ERA-NET: aligning national/regional translational cancer research programmes and activities

HCO 9—2014: ERA-NET: systems medicine to address clinical needs

HCO 10—2014: ERA NET: rare disease research implementing IRDiRC objectives

HCO 11—2015: ERA-NET: collaboration and alignment of national programmes and activities in the area of brain-related diseases and disorders of the nervous system

HCO 12—2015: ERA-NET: antimicrobial resistance

HCO 13—2015: ERA-NET: cardiovascular disease

HCO 14—2014: Bridging the divide in European health research and innovation

HCO 15—2014: Mobilization and mutual learning action plan

HCO 16—2014: National Contact Points

Further, it is highly recommended to analyze systematically the proposals which have been approved and disapproved within “Horizon 2020” with follow-up evaluation of the impacts of corresponding projects which have received financial supported from the EU. EPMA consortium is requested to coordinate these activities at the EU level.

### Endnote

^a^Calls with potential relevance for diabetes care are in italics.

## Competing interests

The authors declare that they have no competing interests.

## Authors' contributions

All authors contributed equally. All authors read and approved the final manuscript.
